# Analysis of Melting Phenomena of the Alkali Halides: What Causes the Low Melting Points of the Lithium Halides?

**DOI:** 10.1002/jms.70065

**Published:** 2026-05-13

**Authors:** Lona Zeneyedpour, Peter C. Burgers

**Affiliations:** ^1^ Department of Neurology, Laboratory of Neuro‐Oncology Erasmus Medical Center Rotterdam the Netherlands

**Keywords:** alkali halides, Lindemann theory, lithium halides, melting, thermodynamic properties, transverse optical frequencies

## Abstract

The alkali halides, like other polar crystals, melt at high temperatures, but the melting points (*T*_*m*_) of the lithium halides are conspicuously lower (by about 300 ± 70 K) than expected from reasonable comparisons with the other alkali halides. For LiCl, LiBr, and LiI, the relatively low *T*_*m*_ values have previously been explained by the facile conversion of the 6:6 coordinated crystal to 4:4 coordination in the liquid. Here we present a different perspective which also includes LiF. After collecting data for a number of bulk properties, it is found that two related properties in particular show a strong correlation with *T*_*m*_, namely, their static dielectric constants (*ɛ*, as was proposed previously from diffusional force theory) and the transverse optical frequencies *υ*_*TO*_ (or *ω*_*TO*_) of the lattice. More precisely, a strong correlation is found between *T*_*m*_ and *μω* ^2^_*TO*_ (previously referred to as the combined force constant, *μ* is reduced mass of an ion pair). This follows from the observation that *υ*_*TO*_ for the lithium halides are significantly smaller than expected from comparative considerations (phonon softening). Related to these lower frequencies is the observation that the Lindemann parameter *ρ* (which is a measure of the vibrational amplitude) is very large (0.33) for the lithium halides, compared with the generally accepted value of 0.22 for a melt, which interestingly is close to the value (0.24 ± 0.02) obtained for the other halides. Thus, we argue that these large vibrational amplitudes cause the lithium halide molecules to “shake” themselves loose at lower‐than‐expected temperatures.

## 1 Introduction

In a recent review on the process of melting, it was concluded that although melting is a familiar physical phenomenon, it may still not be well‐understood [1]. In particular, the structural changes that occur upon melting are not known in detail [2]. Melting point determinations form an important part in many applications of chemistry, physics, and the life sciences, and mass spectrometry has made major contributions towards our understanding of melting. For example, Knudsen effusion mass spectrometry (KEMS) has long been an important experimental tool in physical chemistry [3] to study (near‐) condensed phase/vapor equilibria. More recently, investigation of the melting of small particles or clusters has become possible by ion calorimetric methods developed by Neal et al. [4]. The impulse for such investigations was the earlier finding that the melting points of small particles (more than 1000 atoms) are depressed compared with the bulk material, but investigation of even smaller particles or clusters (of about 100 atoms or less, where properties change rapidly) necessitates size selection by mass spectrometry‐based techniques. That melting point determinations are also important for the development of mass spectrometric tools themselves is shown by a very recent finding that the eutectic mixture LiNO_3_/CsNO_3_ has a surprisingly low melting temperature (134°C in vacuum), which is ideal for the development of a combined cesium/oxygen gun for positive and negative secondary ion mass spectrometry (SIMS) [5].

Nowadays, various thermochemical data sources are available [6, 7, 8, 9], and we are mining these sources for future data analysis. These databases also contain data relating to phase change phenomena, such as boiling and melting. During these preliminary searches, we encountered a long standing observation and this concerns the melting of the alkali halides (MX, mostly M = Li–Cs and X = F–I). Because of their many applications, these seemingly simple substances (including M = Fr and X = At) have been the subject of many publications, but also they serve as models for the development of theoretical chemistry. The peculiar observation is that the melting temperatures of the lithium halides are markedly lower than would be expected from a general comparison, see Table S1. Thus, the melting points of LiX are significantly lower than those of NaX, the opposite of what reasonably might have been expected. It is the purpose of this contribution to shed more light on this phenomenon. This we do by data mining and searching for possible relationships. Our report is organized as follows. First, we briefly discuss relevant differences between melting and boiling. This is followed by a comparison of the melting points of the alkali halides with other bulk properties. Next, a summary follows of previous reports on radial forces, followed by a discussion of lattice charges, energies and frequencies, and of dielectric constants and force constants of the alkali halides in relation to melting. We then discuss Lindemann's theory and evaluate vibrational amplitudes in relation to melting. Finally, we point to the possibility of enhanced dimer formation of the lithium halides at the melting point.

## 2 Results and Discussion

### 2.1 Melting in Relation to Boiling: Temperature, Enthalpy, and Entropy of Transition

**FIGURE 1:**
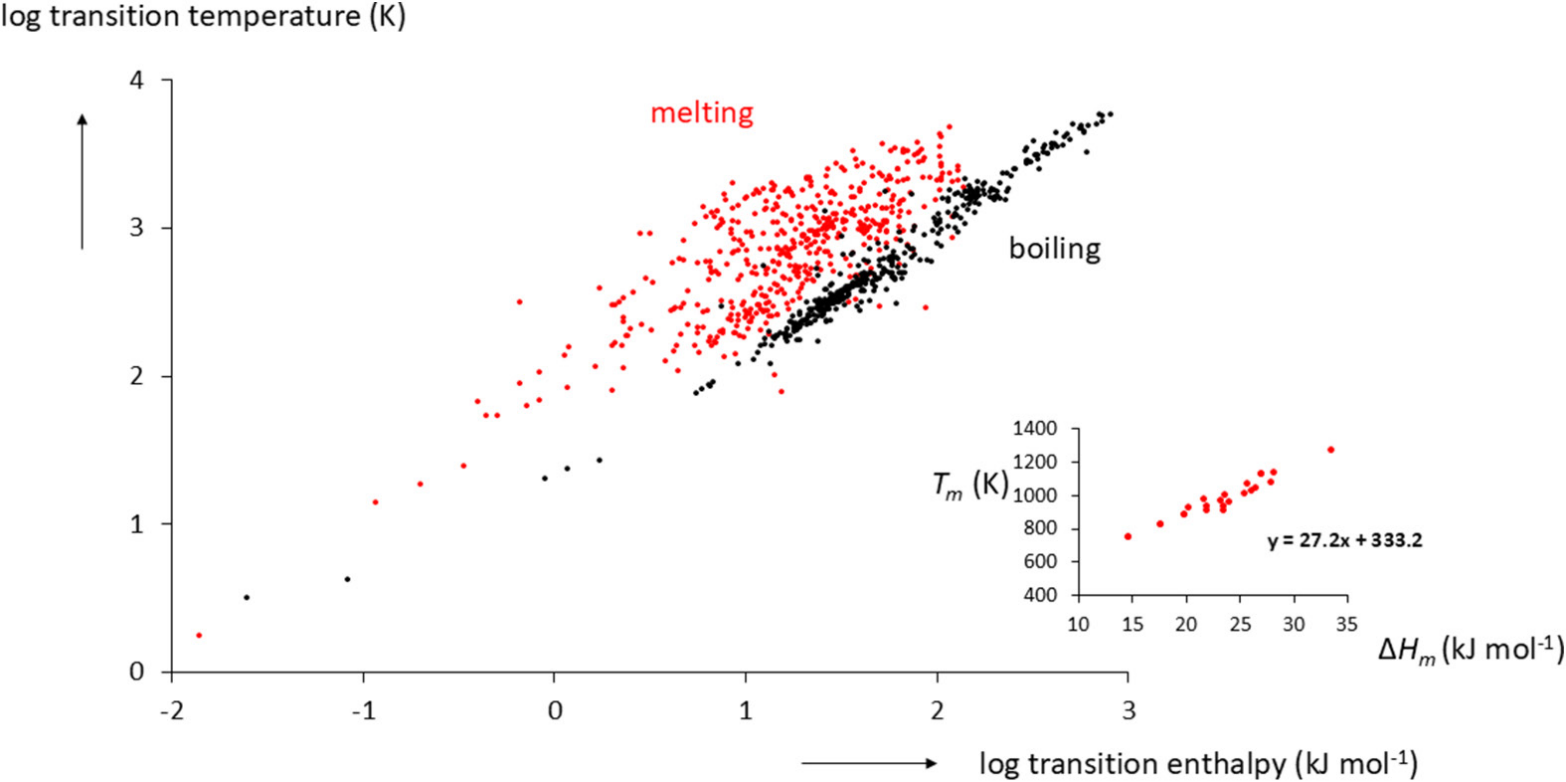
Transition temperature (K) as a function of transition enthalpy kJ mol^−1^ (log‐log plot) of inorganic compounds (# 500). Data from the Yaws data base [6]. Melting: red; boiling: black. Inset: melting temperature (K) versus melting enthalpy (heat of fusion, kJ mol^−1^) for the alkali halides taken from Galwey [2].

We can separate our data files containing enthalpies and entropies of fusion and boiling as well as melting and boiling temperatures, accessed from various databases [6, 7, 8, 9], into organic (# 7000) and inorganic compounds (# 500). Here, we consider only the inorganic substances. In order to plot all data in one figure, we choose a log‐log plot of the melting and boiling points versus the melting and boiling enthalpies, that is, transition temperatures versus transition enthalpies. The result is shown in Figure 1.

The salient features are as follows. The boiling data show a narrow distribution with an average entropy change of Δ*S*_*b*_ = 93 ± 23 J mol^−1^ K^−1^ (from Δ*S* = Δ*H/T*_*m*_). This could be viewed as the inorganic equivalent of the well‐known Trouton rule for organic compounds (for these substances we find a narrower range, Δ*S* = 90 ± 8 J mol^−1^ K^−1^, the Trouton rule). By contrast, the distribution for melting is much wider, Δ*S*_*m*_ = 32 ± 26 J mol^−1^ K^−1^, for which no rule can be extracted, save perhaps a molecular weight dependence. This wide range of entropy values is well‐known for melting and it distinguishes melting from boiling. However, we will show in the following that on occasion, and only for a closely related family of compounds such as the alkali halides MX (but not, e.g., for the alkaline halides MX_2_), Δ*S* values can be very similar within that family. In this respect, the alkali halides are more the exception than the rule. This is shown in the inset of Figure 1 where the melting points of the alkali halides are plotted against their heats of fusion (linear plot) showing an almost linear relationship with an average Δ*S*_*m*_ of 24.1 ± 1.8 J mol^−1^ K^−1^.

### 2.2 Melting in Relation to Other Properties

**FIGURE 2:**
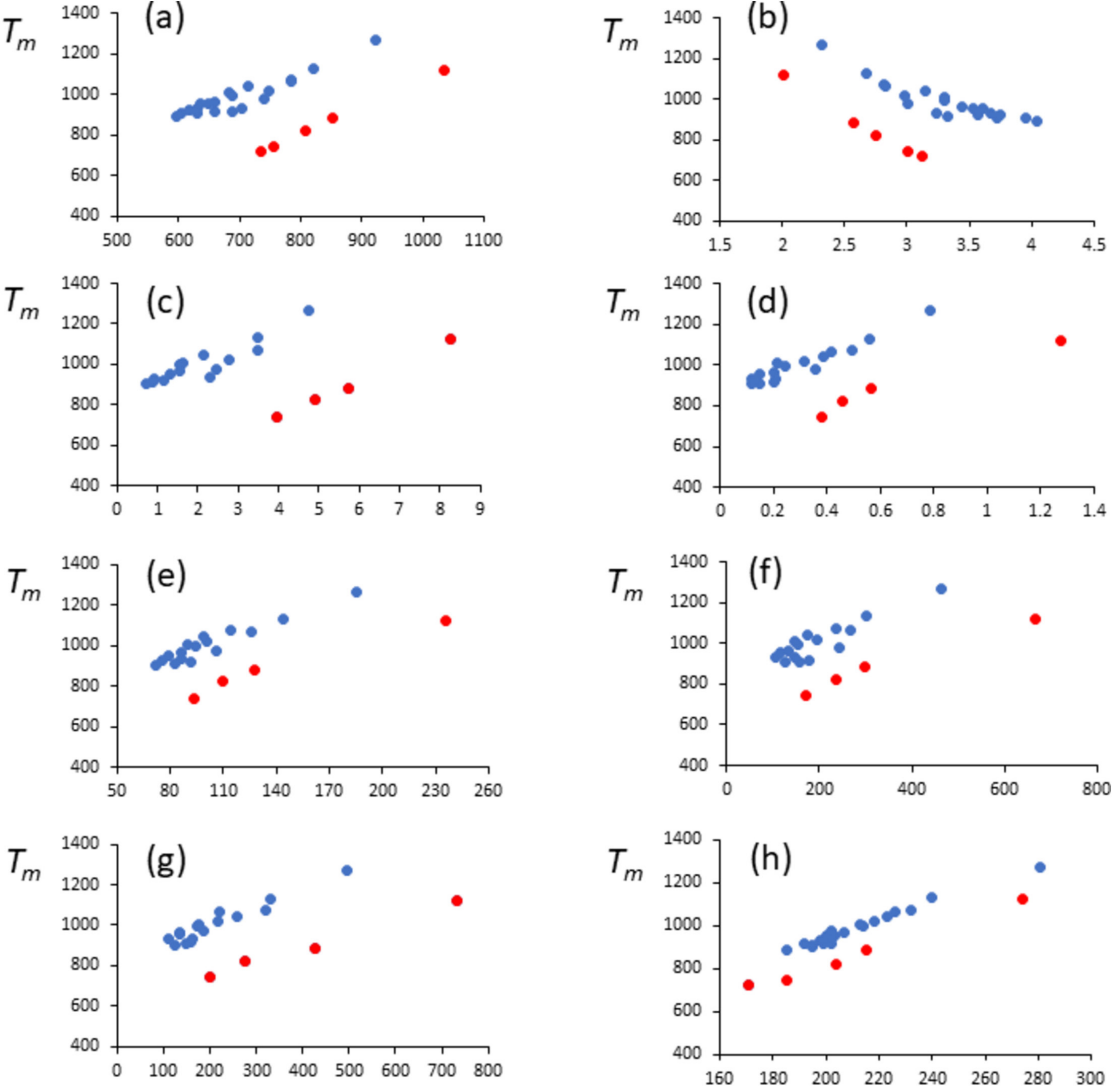
Melting temperatures (K) of the alkali halides as a function of (a) lattice binding enthalpy (*LBE*, kJ mol^−1^) [10]; (b) internuclear distance *r* (Å) [10]; (c) ionic conductivity of the melt (mho cm^−1^) [11]; (d) thermal conductivity above melt (W m^−1^ K^−1^) [12]; (e) surface tension of the melt (mN m^−1^) [13]; (f) bulk modulus (10^8^ N m^−2^) [14, 15, 16]; (g) Debye temperature (K) [17, 18, 19, 20]; (h) sublimation energies (kJ mol^−1^) [10]. Lithium halides in red. Data for the alkali astatides given where available (a,b,h) [10].

When we compare the melting points of the alkali halides to other bulk properties, we find that in almost all cases, the melting points for the lithium halides are much lower than expected from the other properties. In fact, as mentioned by Galwey [2], two subfamilies exist. The bulk properties that we have collected are: lattice binding enthalpies [10], crystal bond lengths [10], ionic conductivities of the melts [11], thermal conductivities of the melts [12], surface tensions of the melts [13], bulk moduli [14, 15, 16], Debye temperatures [17, 18, 19, 20], and sublimation enthalpies [10]. Where possible data for the alkali astatides were included. The data are presented in Figure 2 in which *T*_*m*_ is plotted against each property; Figure 2a is similar to that shown by Galwey [21]. It is clear that in all plots, the red data points of the lithium halides lie below those for the other alkali halides, and thus, of all the properties collected, only the *melting points* of the lithium halides are depressed, not the other properties. (A close examination of Figure 2 reveals a subseries in the blue data points of all graphs, except [h] corresponding to the alkali fluorides [NaF, KF, RbF, and CsF], but this was not further investigated.) From these data, we can estimate that, on average, *T*_*m*_ of the alkali halides are depressed by about 300 ± 70 K. The question is: what causes this reduction in *T*_*m*_?

### 2.3 Radial Forces, Dielectric Constants, Bulk Modulus, Surface Tension, and Melting Points

Neumann [22, 23, 24, 25, 26] and Bosi [27] consider melting of ionic crystals as a process where Coulombian attraction counters a repulsive diffusional force associated with Brownian movement. These authors assume an apparent diffusional driving force *f*_*r*_ = *T* (d*S*/d*r*)_T_ … (**1**), which causes two microscopic spherical particles in a liquid to wander away from each other by means of a three‐dimensional “random walk.” This force is responsible for an increase of the distance *r* between the particles owing to the tendency towards maximum entropy *S*. The first and second law of thermodynamics states d*U* = *T*d*S* − *p*d*V*. If it is assumed that the process is isothermal and reversible (d*U* = 0) then *p* = *T* (d*S*/d*V*) … (**2**). Using pressure = force/area leads to Equation (1). The entropy arises from the number of configurations (*W*) possible for the system, where one particle is fixed at the origin and the other particle moves freely a distance *r* away. The entropy of a particle at a distance *r* from a fixed point is given by Boltzmann's equation, *S* = *k*.ln*W* and *W* is proportional to 4π*r* ^2^. Thus *f*_*r*_ = *T*(d*S*/d*r*) = *T*.(d(*k*.ln*W*)/d*r*) = (*kT*).(1/*W*).(d*W*/d*r*) = 2*kT*/*r*.

The same expression (*f* = 2*kT*/*r*) can be derived from the Helmholtz free energy *A* as follows [25]. From the thermodynamic definition of pressure *p* = −(d*A*/d*V*)_T_ … (**3**), one derives for the force *f*_*a*_ = −(d*A*/d*r*)_T_. Because *A* = −*kT*.ln*Z*, where *Z* is the rotational partitioning function, *f*_*a*_ = *kT*.d (ln*Z*)/d*r* = (*kT*).(1/*Z*).(d*Z*/d*r*). Now, *Z* = (8π^2^ *kTmr* ^2^)/*h* ^2^ and so *f*_*a*_ = 2*kT*/*r*. Thus, *f*_*r*_ and *f*_*a*_ are the same because both *W* and *Z* are of the same function with respect to *r*, namely, *r* ^2^, that is, *W* ∝ *r* ^2^ and *Z* ∝ *r* ^2^. We mention the derivation using the Helmholtz free energy for the following reason. It should be realized, that although Equation (3) (as is found in many textbooks) follows from a characteristic thermodynamic function (namely, *A*(*T*,*V*)), Equation (2) does not. That is to say, from a knowledge of the function *E* = f(*T*,*V*), we cannot find the *V*‐dependence of *S* as is needed. However, for the melting process, the definition of pressure as in Equation (2) (in addition to Equation 3) seems justified. It has further been mentioned [28] that a radially constrained mass point has only two degrees of freedom, and therefore, according to the equipartition theorem, its mean kinetic energy (½*mv* ^2^) is equal to *kT* (as opposed to 32 *kT*) and so the centrifugal force will be *f*_*c*_ *= mv* ^2^/*r* which also equals 2*kT*/*r*.

**FIGURE 3:**
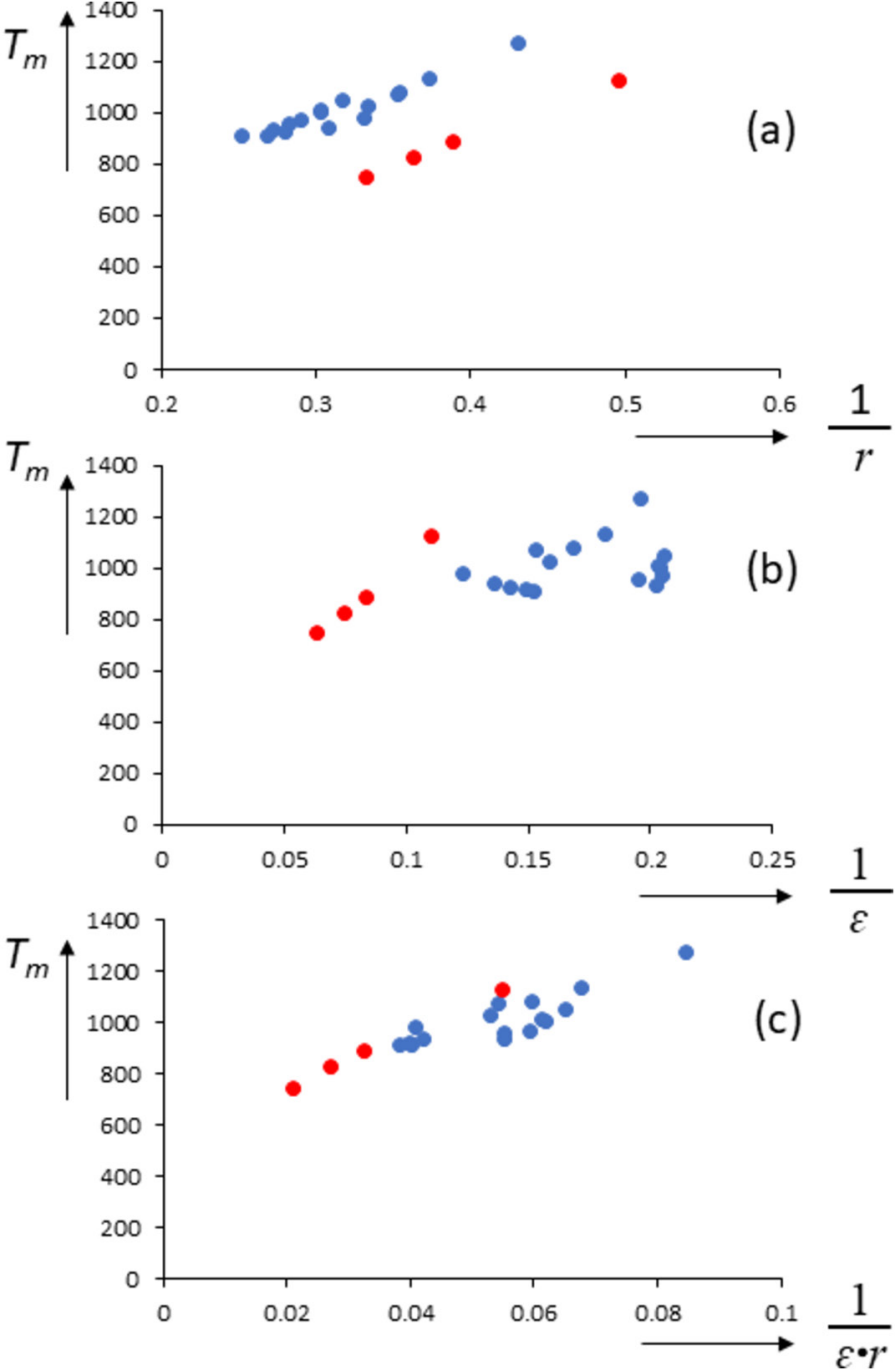
Melting temperatures (K) of the alkali halides as a function of: (a) 1/*r*; (b) 1/*ɛ*; (c) 1/*rɛ*. *r* is internuclear distance (Å), and *ɛ* is dielectric constant (ref). Lithium halides in red.

At the melting point, *T* = *T*_*m*_, the diffusional driving, repulsive force *f*_*r*_ is balanced by the attractive Coulombian force *f*_*c*_ = Z_+_Z_−_ *e* ^2^/(4π*ɛ*_*0*_^.^ *ɛ* ^.^ *r* ^2^) (in SI units [29]) where *ɛ*_*0*_ is the permittivity of vacuum and *ɛ* is the relative dielectric constant of the dielectric medium of the melt. From *f*_*r*_ = *f*_*c*_, it follows *T*_*m*_ = *e* ^2^/(8π*ɛ*_*0*_^.^ *k* ^.^ *ɛ* ^.^ *r*) … (**4**) or *T*_*m*_ = 8.355.10^4^/ *ɛ* ^.^ *r* … (**5**), *T*_*m*_ in K and *r* in Å, Z = 1 for the alkali halides. Interestingly, March et al. [30] have shown that *T*_*m*_ correlates well with the Coulombian energy *e* ^2^/*ɛ* ^.^ *r* for the lithium halides and LiH (but see later for a discussion of *ɛ* (LiI)). Indeed, Equation (5) already points to an intriguing possibility as to why the melting points of the lithium halides are depressed: They possess significantly larger dielectric constants than the other alkali halides. Thus, by plotting *T*_*m*_ versus 1/*ɛ* ^.^ *r*, we can already see that the lithium halides move closer to the other data points as opposed to plots using only 1/*r* or 1/*ɛ*, see Figure 3.

**FIGURE 4:**
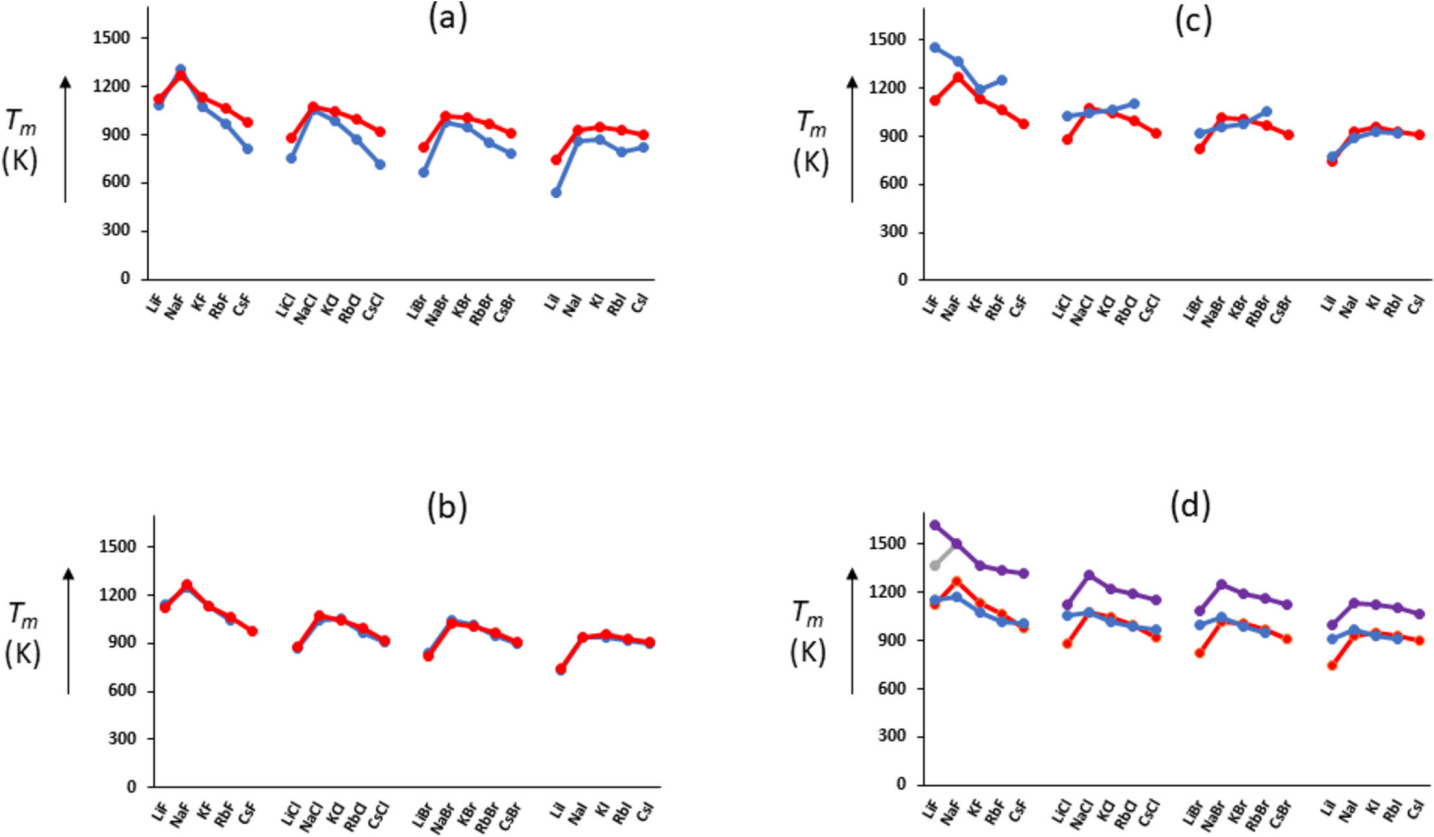
Comparison of calculated (blue) and experimental (red) melting temperatures (K) of the alkali halides: (a) calculated from Equation (7); (b) calculated from Equation (11) using Harrison potential function [14]; (c) calculated from Equation (11) using Born–Mayer exponential potential function [33]; (d) from molecular dynamics (MD) calculations, top data points (purple) Scheiber and Patey [34], bottom data points (blue) from Walz and Van der Spoel [35].

However, inserting the known values of *ɛ* of the alkali halides at 298 K (*ɛ*_*298*_) in Equation (5) leads to *T*_*m*_ values that are too high for all alkali halides. Neumann [23] and Bosi [27] point out that we should really use *ɛ* for the melt (*ɛ*_*melt*_), as opposed to *ɛ*_*298*_, but unfortunately, such values are not known. However, these authors point out that *ɛ* for the melt can be derived from the lattice binding enthalpy (*LBE*) and the melting enthalpy (Δ*H*_*m*_) according to *ɛ*_melt_ = *LBE*/Δ*H*_*m*_ … (**6**). These *ɛ*_*melt*_ values are about factor 3–5 larger than *ɛ*_*298*_, but this may well be the case as studies have shown *ɛ* to increase sharply at elevated temperatures for selected alkali halides, for example NaCl [31, 32]. Equation (5) now becomes *T*_*m*_ = 8.355.10^4^.(Δ*H*_*m*_)/(*LBE* ^.^ *r*) … (**7**). When we take the known values of *LBE* and *r* at 298 K and the measured values of Δ*H*_*m*_, we already achieve reasonable agreement with experiment, see Figure 4a. (For illustrative purposes, the data are grouped for common anions, red = experiment.) In particular, it is found that the melting points of the lithium halides are calculated to be relatively low, as observed for each halogen anion group, leading inverted U‐shaped curves. Because *LBE* and *r* show no particular deviations among the alkali halides, we conclude that the low melting points of the lithium halides are related to their low Δ*H*_*m*_ despite the fact that in general *T*_*m*_ is not very well correlated with Δ*H*_*m*_, see Figure 1. In fact, because *LBE* and *r* are inversely related, then according to Equation (6), *T*_*m*_ will be proportional to Δ*H*_*m*_. Experimentally, *T*_*m*_ is found to be proportional to Δ*H*_*m*_ but not directly proportional, see Figure 1, inset.

Support for Neumann's and Bosi's radial force, *f*_*r*_ = 2*kT*/*r*, comes from a relation between the surface tension (*σ*_*m*_) and melting points of the alkali halides. Aqra [36] derives the equation *σ*_*m*_ (Nm^−1^) = 1.35(*kT*_*m*_ *r*/*V*_*m*_) … (**8**), where *V*_*m*_ is the molecular volume and 1.35 is an empirical fitting parameter. If we take Neumann's equation for the surface tension for the alkali halides, *σ*_*m*_ = *e* ^2^/{(4π*ɛ*_*0*_)(π*r* ^3.^ *ɛ*)} … (**9**), and then eliminate *ɛ* from Equations (4) and (9), we obtain (using *V*_*m*_ = 2*r* ^3^): σ_m_ = (4/π)(*kT*_*m*_ *r*/*V*_*m*_) … (**10**) where the factor is now predicted to be 4/π = 1.27, close to the empirically derived value (1.35). Thus, the experimentally derived factor above is, in fact, predicted by theory. This, we argue, lends great support for Neumann's and Bosi's equation for the diffusional radial force *f*_*r*_ = 2*kT*/*r*. Note that Equation (10) also predicts that surface tensions for the alkali halides are much smaller (by a factor of 3) than those predicted from Dayal's equation [37] derived for organic molecules, as is observed experimentally.

Its success notwithstanding, Equation (7) has come under scrutiny as it invokes only Coulombian forces. In the studies by Liu and Chen [14], Singh [38], and Chauhan and Singh [33], the Coulombian forces are replaced by interionic forces (*f*_*i*_) derived from lattice potential energies, *φ*. The radial force is now evaluated from Equation (1) not by calculating the entropy, but by eliminating it using Maxwell's equations. Equation (1) is first written as a volume derivative using *V* = ⋇*r* ^3^, where ⋇ is the geometrical factor (= 2 for nacl and 1.54 for cscl structures, we use non‐capitals to denote structure type), *f*_*r*_ = *T* (d*S*/d*r*)_T_ = 3⋇*r* ^2^ *T*(d*S*/d*V*)_T_. Then, by using Maxwell's equation (d*S*/d*V*)_T_ = (d*P*/d*T*)_V_ and by using (d*P*/d*T*)_v_ (d*T*/d*V*)_P_ (d*V*/d*P*)_T_ = −1, we get (d*P*/d*T*)_V_ = −(d*P*/d*V*)_T_ (d*V*/d*T*)_P_ = [−*V* (d*P*/d*V*)_T_].[(1/*V*).(d*V*/d*T*)_P_] = *B* ^.^ *α*, where *B* is the isothermal bulk modulus or incompressibility (= −*V* (d*P*/d*V*)_T_) and *α* is the thermal expansivity (= (1/*V*) (d*V*/d*T*)_P_), which are experimentally available. Hence *f*_*r*_ = (3⋇*r* ^2^ *αB*)*T*. This force is then equated to (d*φ*/d*r*) at the melt (*r* = *r*_*m*_), leading to *T*_*m*_ = (1/3⋇*r* ^2^ *αB*)(d*φ*/d*r*)_r = r,m_ … (**11**). Using this procedure, much better agreement is found, see Figure 4b. For comparative purposes, results from MD calculations are also shown [34, 35]. We should mention that the Coulombian forces calculated from *f*_*c*_ = *e* ^2^/(4π*ɛ*_*0*_ *ɛr* ^2^) and the interionic forces calculated from *f*_*i*_ = d*φ*/d*r* differ significantly (by more than a factor 10) as do the radial forces calculated by *f*_*r*_ = 2*kT*/r and *f*_*r*_ = (3⋇*r* ^2^ *αB*)*T*, see Table 1 using NaCl as an example, but surprisingly the calculated melting points are in reasonable agreement with experiment.

**TABLE 1:** Calculated radial forces (*f*_*r*_ = *T* (d*S*/d*r*)), Coulombian forces (*f*_*c*_), interionic forces (*f*_*i*_), and melting temperatures (*T*_*m*_) of NaCl.

Neumann/Bosi		Liu/Singh	
*f*_*r*_ = (2 *k*/*r*).*T*	(0.937.10^−13^).*T*	*f*_*r*_ = (3⋇*r* ^2^ *αB*).*T*	(14.7.10^−13^).*T*
*f*_*c*_ = *e* ^2^/(4π*ɛ*_*0*_ *ɛr* ^2^)	0.095.10^−9^	*f*_*i*_ = (d*φ*/d*r*)	1.602.10^−9^
*T*_*m*_ (calc)	1010	*T*_*m*_ (calc)	1090

*Note:* Forces in Newton (N), temperatures in Kelvin (K). *k* = Boltzmann's constant; *r* = bond length (= 2.948 Å [38]); x = geometrical factor (= 2); *α* = thermal expansivity (= 1.19.10^−4^ K^−1^ [38]); *B* = isothermal bulk modulus (= 2.37.10^10^ Nm^−2^ [38]). *ɛ* = *LBE*/Δ*H*_*m*_ (= 786/28.0 = 28.1); *T*_*m*_ (exp) = 1074 K.

It may be somewhat surprising and counterintuitive to find that the radial forces calculated by Liu and Chen [14], Singh [38], and Chauhan and Singh [33], *f*_*r*_ = (3⋇*r* ^2^α*B*)*T* would appear to *increase* rapidly with *r*, whereas those from *f*_*r*_ = 2*kT*/*r* decrease with *r* (as could reasonably be expected), but it can be shown from experimental data that the bulk modulus *B* shows an *inverse* direct proportionality with *r* ^3^ leading to a net *r* ^−1^ dependence.

The main conclusion we can draw from the above considerations is that the dielectric constant at melt and the bulk modulus (the reciprocal of compressibility) play an important role in determining the melting points of the alkali halides. It is precisely these properties that also play a role in determining the short‐range interaction force constant (*k*) of the lattice via the dielectric constant, *k*_*s*_ = [(*ɛ* + 2)/(*ɛ*_*∞*_ + 2)]*μω* ^2^_*TO*_ … (**12**) [39], see below, or via the bulk modulus, *k*_*bm*_ = 3⋇*rB* … (**13**) [39, 40] where *μ* is the reduced mass per ion pair, *ɛ*_*∞*_ is the optical dielectric constant (= *n* ^2^, where *n* is the refractive index), *ɛ* is the static dielectric constant and *ω*_*TO*_ is the transverse optical (angular) frequency of the lattice. In many cases, the frequency *υ* is expressed as cm^−1^ and *μ* as u and so *μω* ^2^ (Nm^−1^) = *μ*(2π*cυ*)^2^ *N* ^−1^.10^−3^ = 5.892.10^−5^ *μυ* ^2^ (*υ* in cm^−1^, *c* in cm s^−1^, *μ* in u, and *N* is Avogadro's number). In the following, we show that a strong relation exists between *μω* ^2^_*TO*_ and the melting points *T*_*m*_.

**FIGURE 5:**
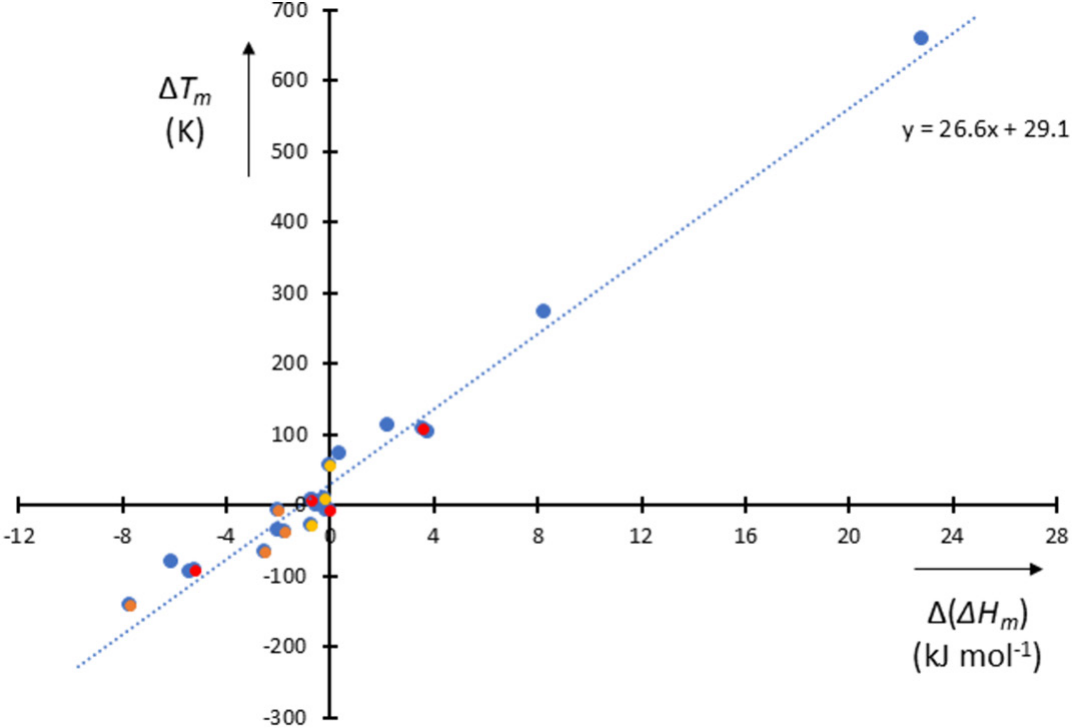
Difference in experimental and calculated melting points Δ*T*_*m*_ (K) versus difference in experimental and calculated melting enthalpies Δ (Δ*H*_*m*_) (kJ mol^−1^). Data from Aragones et al. (blue) [41], DeFever et al. (RIM red, PIM orange) [42], and Zakiryanov et al. (yellow) [43].

A comment concerning *T*_*m*_ calculations from thermochemical properties is warranted. From the inset of Figure 1, from which follows that *T*_*m*_ is proportional to Δ*H*_*m*_, at least for the alkali halides, it would appear that *T*_*m*_ could follow from a theoretical assessment of Δ*H*_*m*_. However, the difficulties associated with such an approach can be appreciated from the slope of the line, which is about 27, which means that for each 1 kJ mol^−1^ increase in Δ*H*_*m*_, *T*_*m*_ goes up by 27 K, and this imposes significant demands on such calculations. This is apparent from the work of Aragones et al. [41], DeFever et al. [42], and Zakiryanov et al. [43]. Their combined results are shown in Figure 5 which shows the *difference* in experimental and calculated *T*_*m*_ values plotted against the *difference* in experimental and calculated Δ*H*_*m*_ values, from which we can see that the slope here, too, is about 27.

### 2.4 Szigeti Charges, Lattice Frequencies, Force Constants, and Melting Points

The static dielectric constant *ɛ* of a crystal is the sum of the electronic and lattice contributions, *ɛ* = *ɛ*_*∞*_ + *ɛ*_*lattice*_ = *n* ^2^ + *ɛ*_*lattice*_. The electronic component is also the optical dielectric constant *ɛ*_*∞*_ and it equals the square of the refractive index, *n* ^2^ [44]. These values are dimensionless. (In some publications, the symbol *ɛ*_*0*_ is used for *ɛ*; we reserve *ɛ*_*0*_ for the permittivity of vacuum, *ɛ*_*0*_ = 8.854.10^−12^ C^2^N^−1^ m^−2^).

**FIGURE 6:**
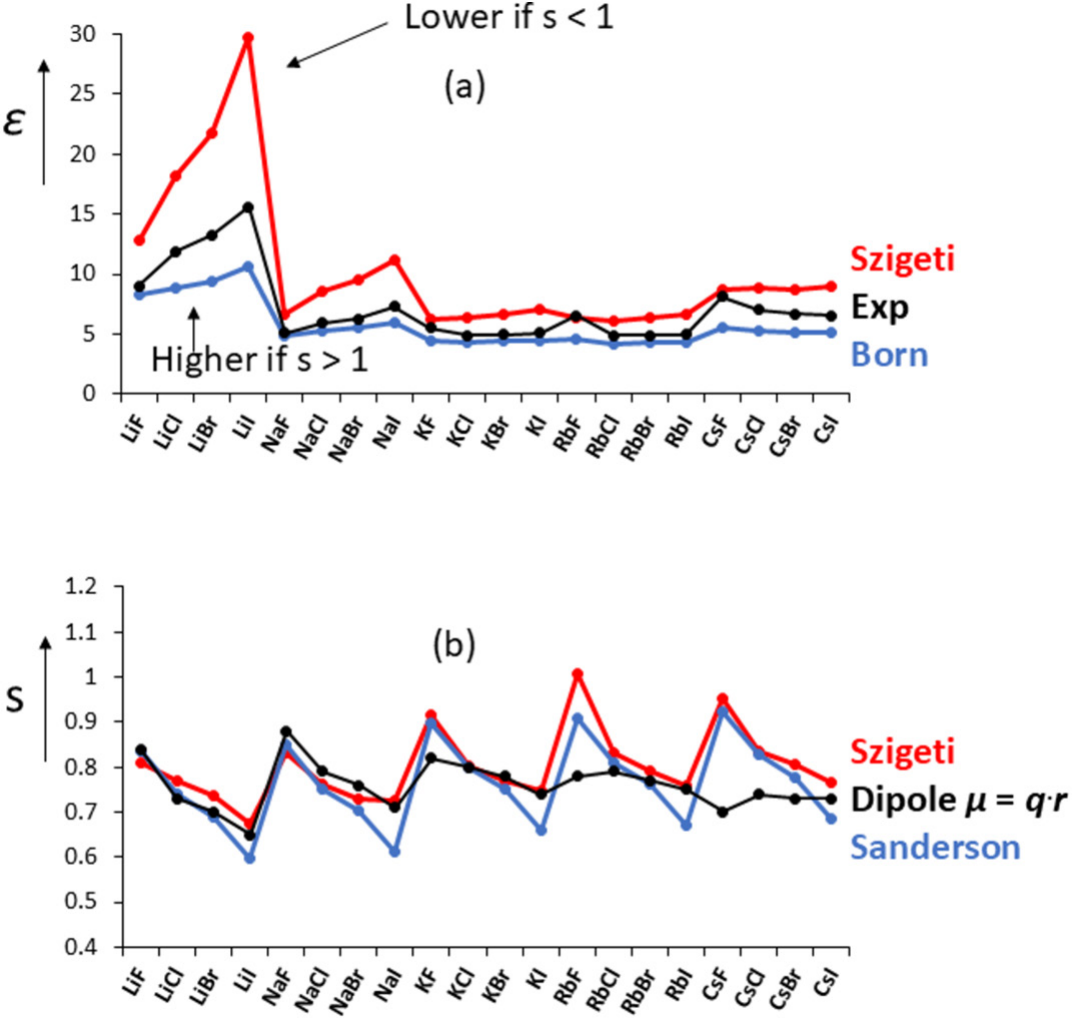
Calculated dielectric constants *ɛ* of the alkali halides: (a) blue: via Born Equation (14); red: via Szigeti Equation (15), experimental values (black) as indicated. Born values can be increased by increasing *s*, whereas Szigeti values can be decreased by lowering s; (b) Szigeti charges of the alkali halides (blue) together with Sanderson charges (gray) of gas‐phase species. Gas‐phase values derived from dipole moments also shown.

The Born equation [45] links the dielectric constant *ɛ* with the transverse optical (angular) frequency: *ɛ* = *n* ^2^ + (Z*e*)^2^/(*ɛ*_*0*_ *Vμω* ^*2*^_*TO*_) … (**14**). *V* is the molecular volume in m^3^, *μω* ^*2*^_*TO*_ is in Nm^−1^, see above, and *e* is the electronic charge in C. For the alkali halides Z = 1; it can be seen that the last term in Equation (14), like the others, is dimensionless. However, it was quickly noted [46] that invariably the values of *ɛ* thus calculated were *less* than the experimental values. Upon realizing that transverse vibrations of the lattice increases atomic polarization, Szigeti [46, 47] derived another equation: *ɛ* = *n* ^2^ + (sZ*e*)^2^/(*ɛ*_*0*_ *Vμω* ^*2*^_*TO*_)}{(*n* ^2^ + 2)/3}^2^ … (**15**), the famous Szigeti equation, where the factor {(*n* ^2^ + 2)/3}^2^ takes into account the contribution from polarization and where the third term is the lattice contribution to *ɛ*. In Figure 6a are shown the calculated *ɛ* values from the Born (blue trace) and Szigeti (red trace) equations together with the experimental values.

It is seen that the Szigeti Equation (15) would appear to over‐correct the *ɛ* values as it gives values for *ɛ* that are now *greater* than those measured as opposed to *lower* values from the Born equation. It is to Szigeti's credit that he introduced the factor s in his formula (see above), which, importantly, turns out to be smaller than 1 (and not greater than 1) to reproduce the experimental *ɛ* values, see Figure 6a. The factor s represents an effective charge, the Szigeti charge; these charges are listed in Table S1. The values for these Szigeti charges are plotted in Figure 6b using the most recent data (red trace). (The equations for calculating the Szigeti and Born charges using *ν* (instead of *ω*) are given in Data S1, see equations in bold.) Nowadays, effective charges are common and accepted features in chemistry, thanks to Szigeti's insight. If effective charges for the alkali halides are calculated from the Born equation (also known as the Born transverse effective charges e*_T_), they are greater, not smaller than unity which would energetically be highly unlikely. Thus, according to Equations (14) and (15), a large static dielectric constant (*ɛ*) arises from a large lattice contribution [44], which in turn is governed by a low transverse optical frequency *ω*_*TO*_. (It is noteworthy that for the lithium and sodium halides, *ɛ* increases as the counter‐ion size increases, with an opposite trend for the other alkali halides, but this was not further investigated).

Comparison with charges obtained for gas‐phase alkali halides prove highly insightful. One procedure to obtain such gas‐phase charges is from the Sanderson electronegativity scheme [48] that, briefly, is based on the premise that when neutral atoms combine, the electronegativities shift to new equilibrium values. So, instead of using electronegativity values from ionization energies and electron affinities, a new scale (the Sanderson scale) is derived empirically. Sanderson's method depends upon a scaling factor, the charges in NaF. These gas‐phase charges are nowadays readily available from ab initio calculations, q (NaF) = + or −0.85 ± 0.04 ([49], average CHG, MKS, and CM5 methods) leading to a scaling factor of 1.84 (compare Sanderson's original factor 2.08). The calculated charges data are also plotted in Figure 6b (blue trace). It is important to stress that the Sanderson distribution is not affected by scaling; the scaling just moves the graph up and down. The similarity between the Szigeti and Sanderson charges is striking. What then emerges is that the charges of the ions do not change from gas phase to solid; thus, it makes no difference whether for example Na^+^ is attached to one Cl^−^ or to six of them, each also attached to Na^+^.

(We note in passing that the Lyddane–Sachs–Teller equation [50] relates the transverse optical frequency *ω*_*TO*_ with the longitudinal optical frequency *ω*_*LO*_ via (*ω*_*LO*_/*ω*_*TO*_)^2^ = *ɛ/n* ^2^ and by using this and rewriting Equation (14), we get a simple relation for the Born transverse effective charge e*: (e*Z*e*)^2^ = *ɛ*_*0*_ *Vn* ^2^ *μ*(*ω* ^2^_*LO*_ − *ω* ^2^_*TO*_) … (**16**), from which it has been concluded that “no matter how one defines ionicity of a crystal, its only manifestation is in the splitting of the longitudinal and transverse optic phonon frequencies at *k* ~ 0” [47, 51]. A similar equation and conclusion may be derived for the Szigeti charge s, (sZ*e*)^2^ = *ɛ*_*0*_ *Vn* ^2^(Δ*ω*)^2^{3/(*n* ^2^ + 2)}^2^.

Another way to calculate ionic charges using differences in electronegativity values (Δ*χ*) is via the procedure of Pauling: *q* = 1 − exp.[−f (Δ*χ*)^2^] … (**17**) [52]. Again, we calculate the calibration factor f (= 0.20) from *q* (NaF) = 0.85. The resulting ionic charges resembles those of Sanderson, but are somewhat smaller, see Table S1.

Yet another way to obtain gas‐phase charges of diatomic molecule is from their experimentally determined dipole moments (*μ*) from the equation *μ* = 4.8032.*q* ^.^ *r* (*μ* in Debye) [53]. These values are also shown in Figure 6b (black trace). It can be seen that notably for the larger cations K^+^, Rb^+^, and Cs^+^, these values deviate significantly from those of Sanderson, especially for RbF and CsF. One reason for this discrepancy is that the mass centers and charge centers in polar diatomic molecules do not coincide [53] and so calculating charges from dipole moments may well not be a correct procedure.

In Figure S1, we have plotted *T*_*m*_ as a function of the Szigeti and Born charges, and it can be seen that *T*_*m*_ becomes larger with increasing Szigeti charges as might reasonably be expected for higher ionicities, but unfortunately, the correlations are weak; hence, if the Szigeti charge can be viewed as a measure of ionicity, then such an ionicity corresponds only weakly to *T*_*m*_.

**FIGURE 7:**
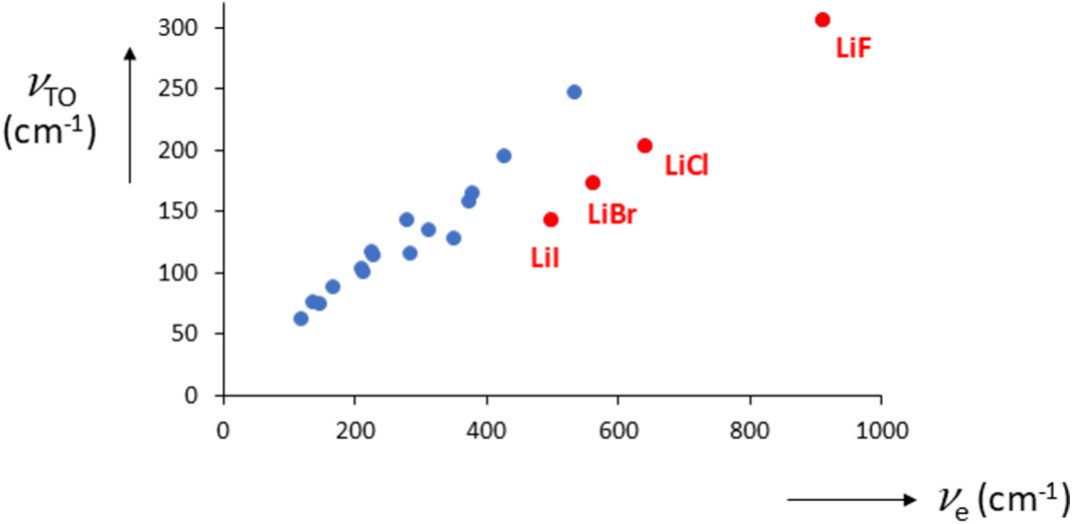
Lattice frequencies (*υ*_*TO*_) versus gas‐phase frequencies (*υ*_e_) for alkali halides; *υ* in cm^−1^. Alkali halides in red.

What is immediately clear from the above is that, for the alkali halide crystals, lattice frequencies, dielectric constants, and atomic charges are all related, and we will discuss them below in some more detail. Solitary gas‐phase alkali halides, like all diatomic molecules, have only one vibrational frequency, *υ*_*e*_ which are accurately known. Solid alkali halides have one dominant characteristic infrared active frequency, the transverse optical frequency *υ*_*TO*_ which, too, are accurately known. These frequencies have also been referred to as characteristic resonant frequencies [54]. When we plot *υ*_*TO*_ against *υ*_*e*_, we see a remarkable effect, see Figure 7.

It appears that the lattice vibrations of the lithium halides are smaller (by about 70 cm^−1^, see below) compared with the values that unite the others. But their melting points are also lower than expected. Are these two effects related? We could not find a unifying relationship of all alkali halides between *T*_*m*_ and *υ*_*TO*_, but a closer examination of the force constants does reveal such a relation.

The force constants (*k*_*g*_) of the solitary gas‐phase alkali halides can be calculated simply from *k*_*g*_ = *μω* ^2^_e_, where *ω*_e_ is the (only) vibrational (angular) frequency. For solid alkali halides, the situation is more complicated. We treat *μω* ^2^ as a separate property (see also [46]) with the force constant unit Nm^−1^. The Born–Huang equation [51], *μω* ^2^_*TO*_ = *k*_*s*_ − {(sZ*e*)^2^/3*ɛ*_*0*_ *V*}/{1‐(*α*_+_ + *α*_−_)/3*ɛ*_*0*_ *V*}, where *α*_+_ ad *α*_−_ are the polarizabilities of the cation and anion, can be rewritten using the Clausius–Mossoti relation, (*α*_+_ + *α*_−_)/3*ɛ*_*0*_ *V* = (*n* ^2^–1)/(*n* ^2^ + 2), to obtain *μω* ^2^_*TO*_ = *k*_*s*_ − (sZ*e*)^2^(*n* ^2^ + 2)/(9*ɛ*_*0*_ *V*) … (**18**), and these equations highlight the various relationships. Rewriting the Szigeti Equation (15), we get (sZ*e*)^2^ = 9*ɛ*_*0*_ *Vμω* ^2^_*TO*_(*ɛ* − *n* ^2^)/(*n* ^2^ + 2)^2^ … (**19**). Substitution of (sZ*e*)^2^ of Equation (19) into Equation (18) gives *k*_*s*_ = [(*ɛ* + 2)/(*n* ^2^ + 2)]*μω* ^2^_*TO*_ (Equation 12, see above), which represents the well‐known short‐range interaction force constant (*k*_*s*_) of the lattice. (Application of the *average* longitudinal‐transverse frequencies, *ω*_*LTO*_, as defined by Baonza et al. [55], leads to the simple equation: *k*_*s*_ = *μω* ^2^_*LTO*_.) Another approach to evaluate force constants is given by Kittel [56]. The positive ions are considered to lie on a set of planes as do the negative ions lying on separate planes leading to interleaved planes. The positive and negative ions are connected by a force constant denoted by *C*. The following equation results 2*C* = *μω* ^2^_*TO*_ … (**20**). Other expressions for force constants can be found [57, 58]. It is of interest to note that in earlier work Raman [59] uses the term operative force constant for *μω* ^2^_*TO*_, whereas Dayal [60] referred to it as combined force constant *D*; more recently, it has been equated (approximately) to the sum of intra‐ and inter‐lattice spring constants [61]. We shall use *D* for the expression *μω* ^2^_*TO*_.

**FIGURE 8:**
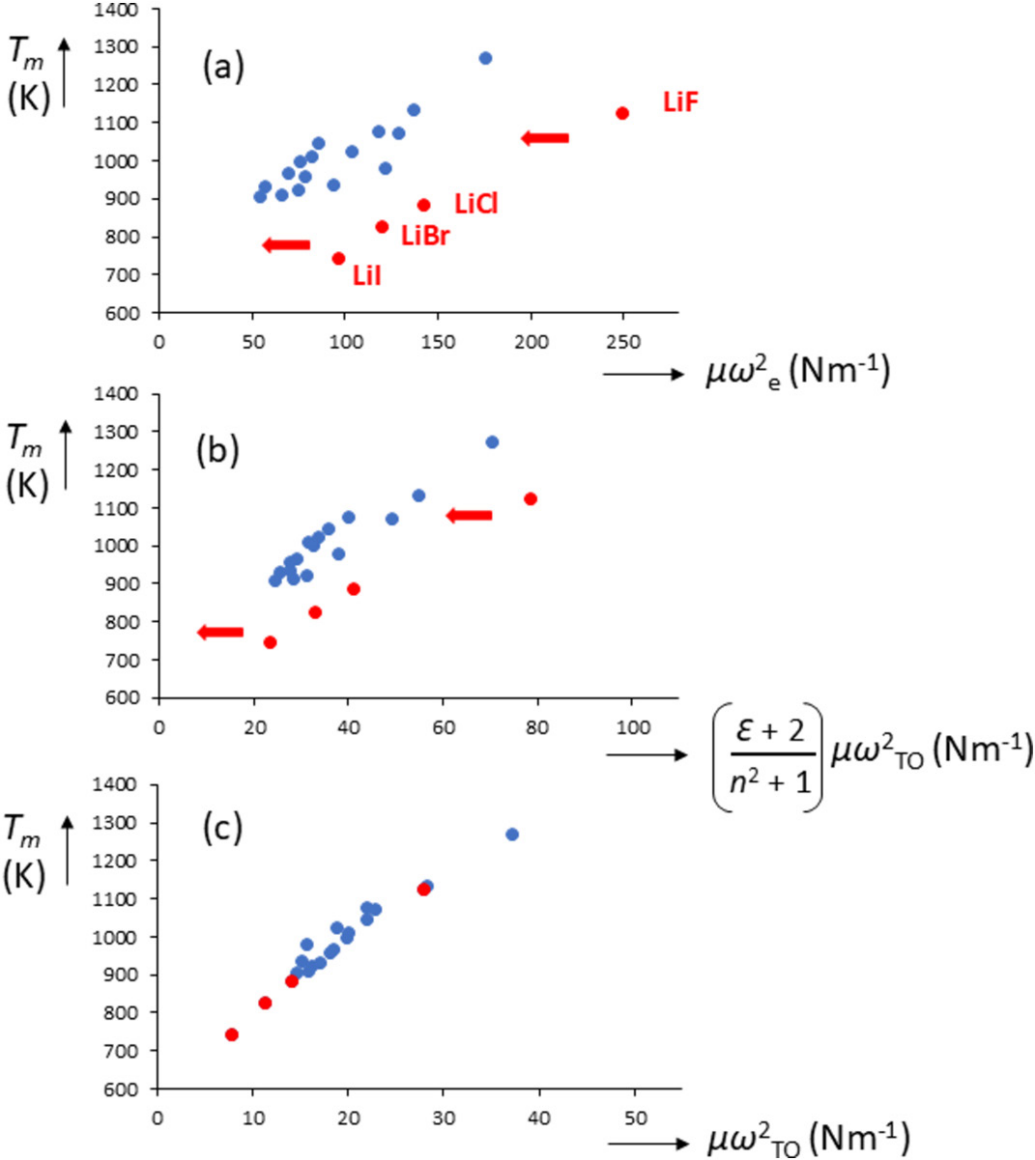
Melting temperature *T*_*m*_ (K): (a) against gas‐phase force constant *μω* ^2^_e_; (b) against short‐range force constant *k*_*s*_ = [(*ɛ* + 2)/(*n* ^*2*^ + 2)]*μω* ^2^_*TO*_; (c) against *μω* ^2^_*TO*_. Lithium halides in red. Note that *k*_*s*_ in subpart (b) can be equated to *μω* ^2^_*LTO*_ as defined by Baonza et al. [55].

Values of *k*_*s*_ and *D* are given in Table S1, together with those derived from *k*_*bm*_ = 3⋇*rB* (Equation 13), as well as those for gas‐phase species, *k*_*g*_. It can immediately be seen that the values of the short‐range force constants *k*_*s*_ and *k*_*bm*_ are very similar. They are also, as might reasonably be expected, much smaller than *k*_*g*_. The values for *D* are even smaller, but more importantly, the *D* values for the lithium halides break the trend observed for the others. For example, *D* (LiF) is smaller than *D* (NaF), which is the opposite of what is found for the others; similar observations pertain to the other lithium halides. This is a direct consequence of the relatively low *ω*_*TO*_ values found for the lithium halides mentioned above. To investigate possible relationships with melting phenomena, we have plotted, in Figure 8, *T*_*m*_ against the obtained force constants. In Figure 8a, it is seen that for *T*_*m*_ versus the gas‐phase *k*_*g*_, the lithium halides do not follow the trend that unites the others, as was the case for many other properties, see Figure 2. For *T*_*m*_ versus the short‐range *k*_*s*_, we see that data points for the lithium halides moving closer to the others (Figure 8b) and for *T*_*m*_ versus the combined force constant *D*, we see that the data coincide and are now unified (Figure 8c). Thus, there appears to be a strong correlation between *T*_*m*_ and *μω* ^2^_*TO*_.

### 2.5 Vibrational Amplitudes and Melting Point, Lindemann's Theory

Lindemann proposed that melting is the result of vibrational instability (as opposed to mechanical instability) in the crystal lattice when, due to their thermal motion, the molecules would just make contact [62]. Lindemann introduced a parameter *ρ* where *ρr*/2 is the maximum elongation of the oscillating atoms from their equilibrium position to touch the neighboring atoms, thus the elongation *ρ* is *relative* to *r*. Lindemann approximates the oscillator energy at *T*_*m*_ by the thermal energy leading to *kT*_*m*_ = 18 (*βρ* ^2^ *r* ^2^) … (**21**), where *β* is the “spring constant” of the oscillator, and *β* = 4π^2^ *mυ* ^2^ = *mω* ^2^, where *m* and *υ* are the oscillator mass and frequency [63]; *k* is Boltzmann's constant. It has been shown [64] that for fcc structures, a typical value of *ρ* = 0.22 (a 22% amplitude increase, above which the molecule “shakes” itself loose) is typical at the melting point. Indeed, the parameter *ρ* is assumed to be independent of *r* and of the substance at the melting temperature; we will show that this assumption does not hold for the alkali halides. (Interestingly, Lindemann also relates *ρ* to the dielectric constant *ɛ*, *ρ* = 1.26[1–1.105(*ɛ* − 1)(*ɛ* + 2)]^⅓^, but this gives very small and even negative values for *ρ*.) Using the values for the transverse optical frequencies, we have calculated *ρ* at *T*_*m*_ according to Equation (21), which upon rewriting and using *β* = *μω* ^2^_*TO*_ (= *D*) gives: *ρ* = {8*kT*_*m*_ */*(*μω* ^2^_*TO*_^.^ *r* ^2^)}^½^ = 0.105.{*T*_*m*_ */*(*μω* ^2^_*TO*_^.^ *r* ^2^)}^½^ … (**22**), *r* in Å, see Table S1. It can immediately be seen that the values for the lithium halides (0.329 ± 0.007) are far larger than those of the others (0.24 ± 0.02). Note that the latter value is close to the generalized value, 0.22, discussed above. It may be concluded that *ρ* values are not constant, not even in one family, and that for the lithium halides, the vibrational amplitudes are at their maxima already at the observed low *T*_*m*_ values.

Lindemann did not calculate melting points, but used the experimental *T*_*m*_ to calculate the vibrational frequencies, assuming that *ρ* is a constant for the alkali halides considered in his study (KCl, KBr, KI, and NaCl, using KCl as reference). Rewriting Equation (22) we get *T*_*m*_ = (8 *k*)^−1.^ *ρ* ^2^ *r* ^2^ *μω* ^2^_*TO*_ = 90.537.*ρ* ^2^ *r* ^2^ *μω* ^2^_*TO*_ … (**23**). The line in Figure 8c (*T*_*m*_ vs. *μω* ^2^_*TO*_) does not go through the origin because of the factor *ρ* ^2^ *r* ^2^ in Equation (23) which varies by a factor of up to 2.8.

From we above, we propose that the low melting points of the lithium halides are caused by their extraordinarily large vibrational amplitudes, in combination with their (relatively) low vibrational frequencies.

We note that, for LiCl, LiBr, and LiI, the relatively low *T*_*m*_ values have been explained by the facile conversion of the 6:6 coordinated crystal to 4:4 coordination in the liquid, which requires less than 3 kJ mol^−1^ [2]. Our results do not invalidate earlier proposals for the low melting points of the lithium halides, but provide an alternative rationalization. Thus, the facile 6:6 to 4:4 interconversion mentioned above may be a consequence of the low vibrational frequencies involved.

### 2.6 Vibrational Frequencies, Force Constants, and Internuclear Distances

**FIGURE 9:**
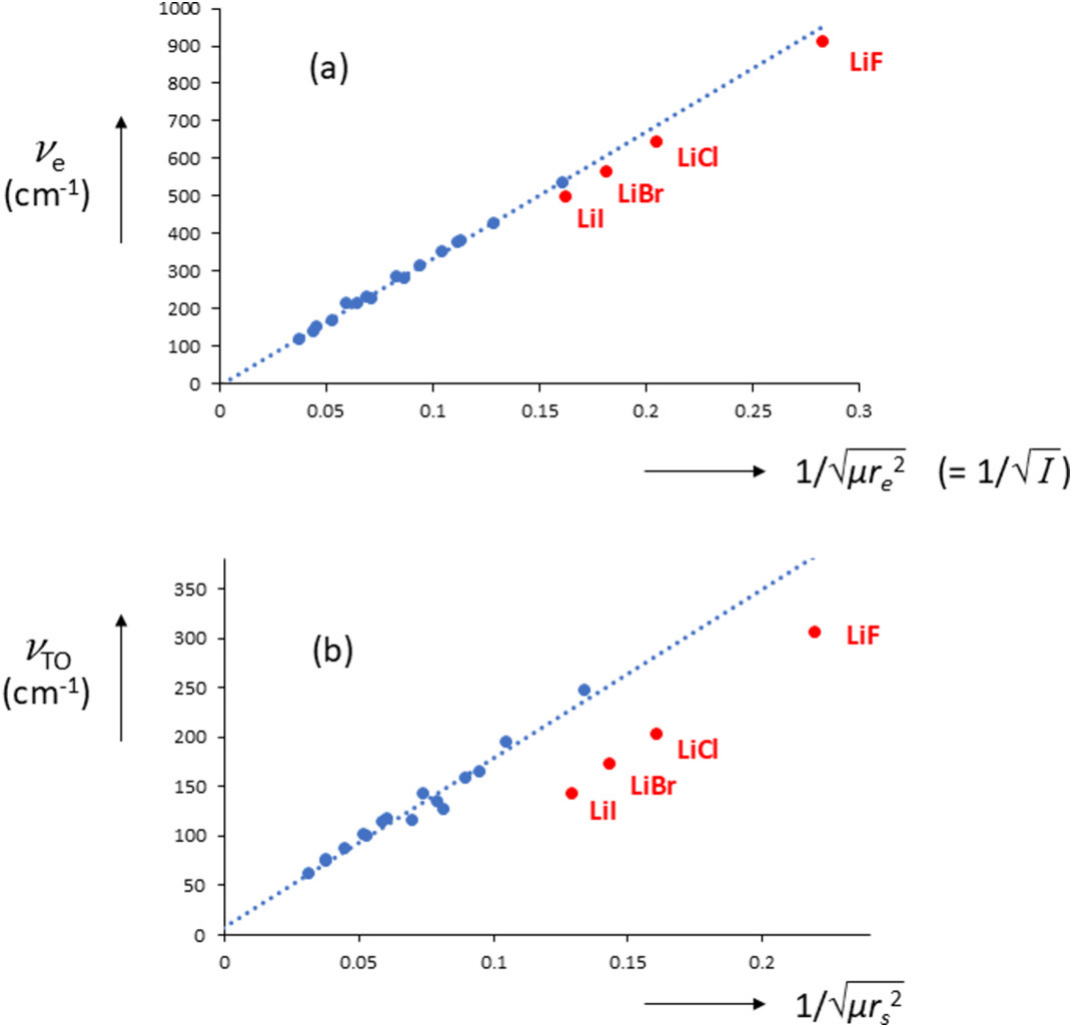
(a) Gas‐phase frequencies as a function of (*μr*_e_^2^)^−½^; (b) transverse optical frequencies against (*μr* ^2^_s_)^−½^. Lithium halides in red.

For gas‐phase diatomic molecules, Badger's rule [65] correlates force constants (*k*) with bond length (*r*) as *k*(*r* − b)^3^ = constant, where b is an empirical parameter. Guggenheimer [66] proposed the relation (*kr* ^c^)/(z_1_z_2_)^½^ = constant, where c depends on the group in the periodic table and where z_1_ and z_2_ are the numbers of electrons in the outer shells of the two atoms (1 for the alkali halides). Zavitsas [67] has found that the relation *υ* = constant.(*μr* ^2^)^−½^ … (**24**) works quite well (equivalently, Zavitsas' equation can be written as: *kr* ^2^ = constant, i.e., c = 2 in Guggenheimer's equation). Interestingly, Equation (24) can be formulated as *υ* = constant.(*I*)^−½^ where *I* is the moment of inertia of the diatomic molecule [68]. We have applied Equation (24) for the alkali halides gas‐phase and solid species, see Figure 9.

For the gas‐phase species (Figure 9a), a linear correlation is obtained going through the origin with the lithium halides lying a little removed from the line that unite the others, but it is a small effect, if any. However, for the crystals (Figure 9b), the lithium halides lie far below the others, showing that their lattice vibrational frequencies are relatively low. From this figure, we can estimate the reduction (in cm^−1^) of *ω*_*TO*_ (phonon softening) for the lithium halides (78, 80, 79, and 86, respectively, for LiF, LiCl, LiBr, and LiI). We note that phonon mode softening has been held responsible for increase in dielectric constants due to lattice strain [69]. A brief discussion of the dielectric constants and lattice energies, in particular those for the lithium halides, follows.

### 2.7 Dielectric Constants of the Lithium Halides and Melting Points

The static low‐frequency dielectric constant *ɛ* of the alkali halides, except that for LiI, are well known and are listed in Table S1. It can be seen that the values for LiF, LiCl, and LiBr are significantly larger than the others. For LiI, a value of 11.03 can be found in various reference works, but this value has been questioned earlier [70], probably because of experimental problems associated with the high hygroscopicity of LiI. The value of 11.03 breaks a trend observed for all the others, for example, *ɛ* for LiI would be smaller than those for LiCl and LiBr. Indeed, from Wang et al. [71] a value of > 13.3 is estimated, while from the work of Lipari et al. [72], a value of 15.4 ensues. The latter value is also in agreement with the value obtained if *ɛ* for LiI would follow *ɛ*_*∞*_ for LiI (Figure S2). By such comparisons, we propose *ɛ* for LiI to be 15.5, not 11.03. Interestingly, March et al. [30] conclude, as we do, that the value of 11.03 may be erroneously low. We can see the importance of the dielectric constant (*ɛ*) for the melting points of the lithium and sodium halides from Figure 3c.

Combination of Equations (12) and (13), that is, assuming *k*_*s*_ = *k*_*bm*_, leads to $\omega_{\text{TO}}=\sqrt{\left(\frac{3xr_{s}B}{\mu }\right)\left(\frac{n^{2}+2}{\varepsilon +2}\right)}$ … (25), (x = geometrical factor, *B* = bulk modulus). Because *B* is directly proportional to *r*_*s*_^−3^, we can write *ω*_*TO*_ ∝ (*μr*_*s*_^2^)^−½^{(*n* ^2^ + 2)/(*ɛ* + 2)}^½^. Interestingly, this is very similar to the gas‐phase relation, see above: *ω*_*e*_ ∝ (*μr*_*e*_^2^)^−½^, the difference being the term (*n* ^2^ + 2)/(*ɛ* + 2). We can thus write for the ratio: *ω*_*TO*_ */ω*_*e*_ ∝ (*r*_*e*_ */r*_*s*_) {(*n* ^2^ + 2)/(*ɛ* + 2)}^½^. Because *r*_*e*_ */r*_*s*_ is approximately constant, we get *ω*_*TO*_ */ω*_*e*_ ∝ {(*n* ^2^ + 2)/(*ɛ* + 2)}^½^. Thus, because the *ɛ* values for the lithium halides are very large, their *ω*_*TO*_s will be relatively low as will be their melting points *T*_*m*_.

### 2.8 Lattice Energies of the Lithium Halides

The possibility may exist that the lithium halides could undergo a transition from fcc to for example bcc prior to or at melting, thus affecting *T*_*m*_, but there is no evidence for such a transition. Various types of lattice structures have been calculated by ab initio methods. Experimentally, CsCl, CsBr, and CsI have the cscl structure (body centered cube, bcc) with coordination number (CN) 8, and the others adopt the nacl or rock salt structure (face centered cube, fcc) with CN = 6. The salient features of these structures are listed in Table 2 together with those of the zns structure (CN = 4). These parameters define the structures, but not their energies.

**TABLE 2:** Coordination number (CN), geometrical factor (x = *V*_*M*_ */r*_*e*_^3^), number of ion pairs per unit cell (= *V*_*unit*_ */V*_*M*_) and ratio lattice parameter/nearest neighbor distance (*a/r*_*e*_) for cscl, nacl (rock salt), and zns crystal structures.

Structure	CN	x	*V*_*unit*_/*V*_*m*_	*a/r*_*e*_
cscl	8	893 ≈1.54	1	233 ≈1.15
nacl	6	2	4	2
zns (cubic)	4	1693 ≈3.08	4	433 ≈2.31

*Note: V*_*m*_ = molecular volume; *V*_*unit*_ = unit cell volume.

**FIGURE 10:**
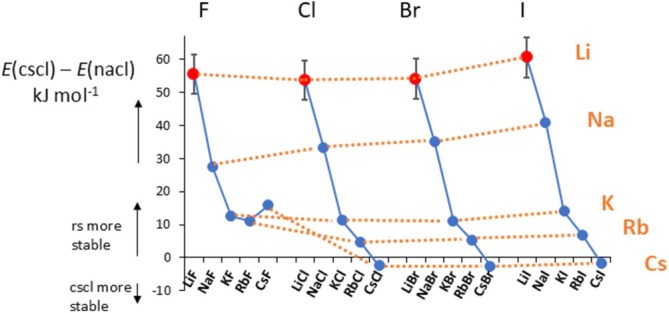
(a) Differences in cesium chloride (cscl) and rock salt (rs) lattice energies at the PBE D2 [74] and PBE D3 [73] levels of theory.

In searching for energetic information which would cover both nacl and cscl structures for all alkali halides, we found two articles which combined yields information for all alkali halides. The closest ab initio methods which we can compare with each other are PBE D3 by Scheiber and Patey [73] for the lithium halides and PBE D2 for the other alkali halides by Solovyeva and Anatole von Lilienfeld [74]. The combined results are shown in Table 3 and Figure 10, which for discussion purposes are sorted in groups of common anions (*x*‐axis). Thus for example, it can be seen that both experiment and theory predict the cscl structure of CsCl to be only about 2–3 kJ/mol more stable than the nacl structure, showing excellent agreement between theory [73, 74] and experiment [75, 76, 77].

**TABLE 3:** Difference of cscl and rock salt lattice energies (kJ mol^−1^).

	F	Cl	Br	I	Method	References
Li	55.5	53.7	54.1	60.5	PBE D3	Scheiber [73]
Na	27.6	33.2	35.1	40.9	PBE D2	Solovyeva [74]
K	12.5	11.3 (9.0)	11.2 (8.4)	14.8 (8.4)	PBE D2	Solovyeva [74]
Rb	11.2	4.6 (3.6)	5.4 (3.0)	6.8 (3.6)	PBE D2	Solovyeva [74]
Cs	15.8	−2.3	−2.5	−1.5	PBE D2	Solovyeva [74]
		−2.96 ± 0.08 −2.89 ± 0.12				Exp: Arell [75] Exp: Pöyhönen [76]

*Note:* Values in brackets: Tosi and Fumi [77].

The *y*‐axis in Figure 10 represents *E* (cscl structure)–*E* (nacl structure), thus positive numbers indicate that nacl is more stable, and negative numbers indicate that cscl is more stable. An earlier conclusion [77] is immediately evident from this picture: The stability of the nacl structure of an alkali halide relative to the cscl structure decreases as the “size” of the *cation* increases, and it is greatest for the lithium halides. That the cscl structures are stable by a small amount only (a few kJ mol^−1^) is in agreement with the finding that CsCl undergoes transition to fcc below the melting point and CsBr and CsI undergo these transitions at *T*_*m*_ [78]. Thus, it would appear that especially the lithium halide fcc structures are quite stable with respect to the bcc structures. However, it has also been shown that wurtzite structures lie close to the rock salt structures for the lithium halides, but we have not addressed the possibility of rock salt to wurtzite transition.

### 2.9 Radial Distribution Functions and Possible Dimer Intermediacy

**FIGURE 11:**
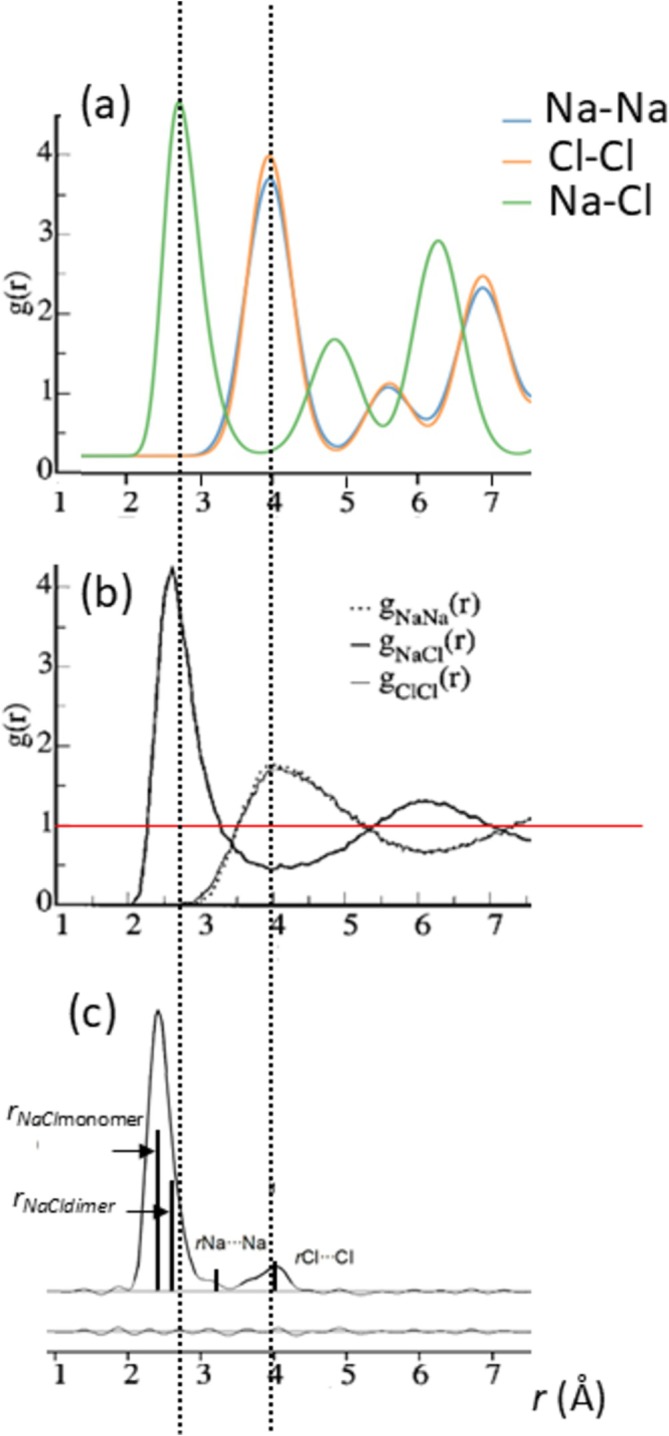
Radial distribution functions for NaCl: (a) at 298 K (from https://vasppy.readthedocs.io/en/latest/examples/rdfs.html); (b) at 1066 K [90], reproduced with the permission of AIP Publishing; (c) vapor at 943 K [86], reproduced with the permission of the American Chemical Society; *T*_*m*_ = 1073.85 K.

We briefly discuss the possible participation of dimeric structures in the melting process. We do this for two reasons. The first is that different techniques have shown that at elevated temperatures, the vapor of alkali halides contain dimers, but that in particular for the lithium halides, large amounts of dimers have been found by mass spectrometric, molecular beam [79], and photoelectron spectroscopy studies [80]. For example, velocity distribution experiments and photoelectron spectroscopy reveal that LiBr vapor at approximately 850 K (well below *T*_*m*_ = 1020 K) consists for 40% of dimers [80, 81] (with also some trimers) with smaller amounts for the other alkali halides (~20% [82, 83, 84, 85, 86]. Similarly LiF may contain 40%–60% dimers [87]. Valuable information concerning the melting process can also be extracted from neutron diffraction experiments, in particular from radial distribution functions (RDF, [88]), which give the probability of finding a particle at a certain distance from a given particle; the RDF describes how the atomic density varies as a function of distance from the atom and is given by *g(r)* = *n(r)*/(4π*r* ^2^.Δ*r* ^.^ *d*), where *n(r)* is the number of atoms at a distance *r* within a shell of thickness Δ*r* and *d* is the average number density [89, 90]. RDF's for NaCl crystals at the melt and for its vapor are shown in Figure 11. These data are from different sources, so we have aligned the *x*‐axes. For fcc structures the *x*‐axis data points of such plots correspond to *r*.√*n*, where *n* is an integer and *r* is the bond length (i.e., for *n* = 1). The horizontal red line is the RDF for an ideal gas (RDF = 1). Figure 11a can be obtained from freely available software (https://vasppy.readthedocs.io/en/latest/examples/rdfs.html).

**FIGURE 12:**
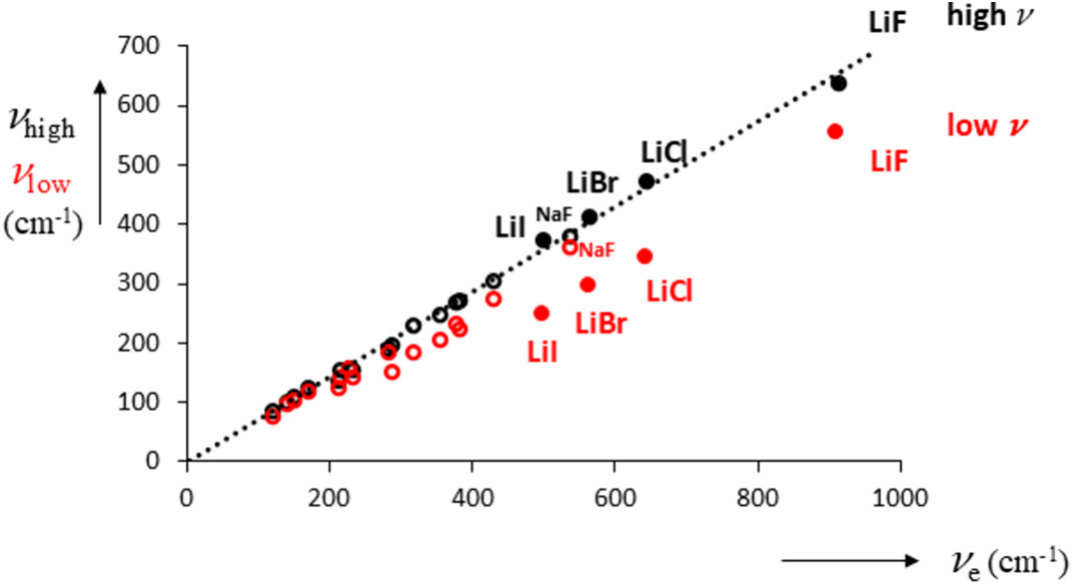
Low (red) and high (black) frequency stretching vibrations of alkali halide dimers as a function of monomer stretching frequency (in cm^−1^).

This figure shows Na–Cl distances as well as Na–Na and Cl–Cl distances in the crystal. Upon melting (Figure 11b), some structure is lost, oscillating away to RDF = 1, but note that the maxima of like–like coincide with the minima of Na–Cl and vice versa, showing that some structure is retained at melt [91]. In addition the RDF for the vapor (Figure 11c) can be readily interpreted as arising from a mixture of monomers and dimers [80] (about 20% dimer). The second reason to discuss dimers is that they show frequency stretching vibrations that are relatively low for the lithium halides as was the case for the crystal structures. Briefly, because the dimer structure is not square (D_4h_), but a rhombus with D_2h_ symmetry, there are two symmetrical stretch vibrations, B_2u_ and B_3u_, which are available in the literature. Unfortunately, there does not appear to be a consensus as to the correct notation (thus, B_2u_ and B_3u_ are interchanged in McCaffrey et al. [86] and Martin and Schaber [92], see also Mwanga et al. for CsF [93] and CsCl [94] compared with Martin and Schaber [92]), and so to avoid confusion, we simply label the frequencies as “high” and “low.” In Figure 12, we plot the dimer frequencies *υ*_high_ and *υ*_low_ as a function of the monomer frequency *υ*_e_. We can see that a considerable frequency splitting is present but only to a significant extent for the lithium halides, see also Table S1. This splitting continues for higher clusters [95], for example, for (LiF)_4_ with T_d_ symmetry. Thus, only the lithium halide dimers show a significant lowering of one of its stretching vibrations, which we argue continues onwards to higher clusters and to the crystal structures, as observed.

Thus, it would be of interest to ascertain whether enhanced formation of dimeric structures for the lithium halides contributes to their lower melting points.

### 2.10 Extension to Alkaline Dihalides MX_2_

**FIGURE 13:**
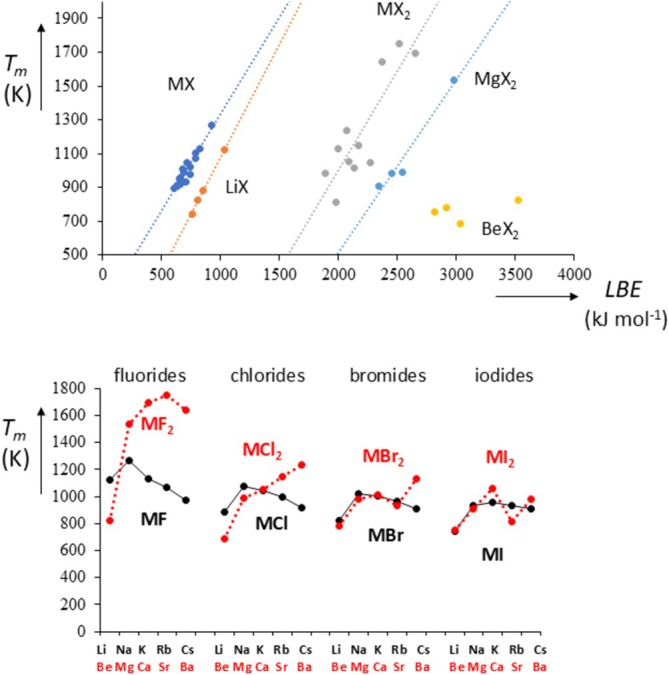
(a) Melting points versus lattice binding energies (*LBE*) for alkali halides MX and alkaline dihalides MX_2_. LiX forms a subset as do BeX_2_ and MgX_2_; (b) Comparison of melting points of alkali halides (black) and alkaline dihalides (red).

Figure 2a shows *T*_*m*_ as a function of the lattice binding enthalpy (*LBE*) from which it can be seen, and as discussed above, that the lithium halides belong to a subset having low *T*_*m*_. A similar plot can be made for the alkaline dihalides MX_2_ [96], see Figure 13a. We can now see that the data can be divided into three sets, with MgX_2_ and BeX_2_ showing the anomalous behavior discussed for LiX. Thus, it would appear that the lowering of *T*_*m*_ for the smaller metal ions now not only occurs for the beryllium crystals but also for the magnesium salts; this can be most easily seen from Figure 13b, which schematically plots the *T*_*m*_ data for the alkali halides and alkali dihalides. The effect is particularly strong for the fluorides and chlorides. Whether this is due to the importance of increased polarization effects for MX_2_ compared with MX remains to be seen [91].

### 2.11 Very Early Work Revisited

**FIGURE 14:**
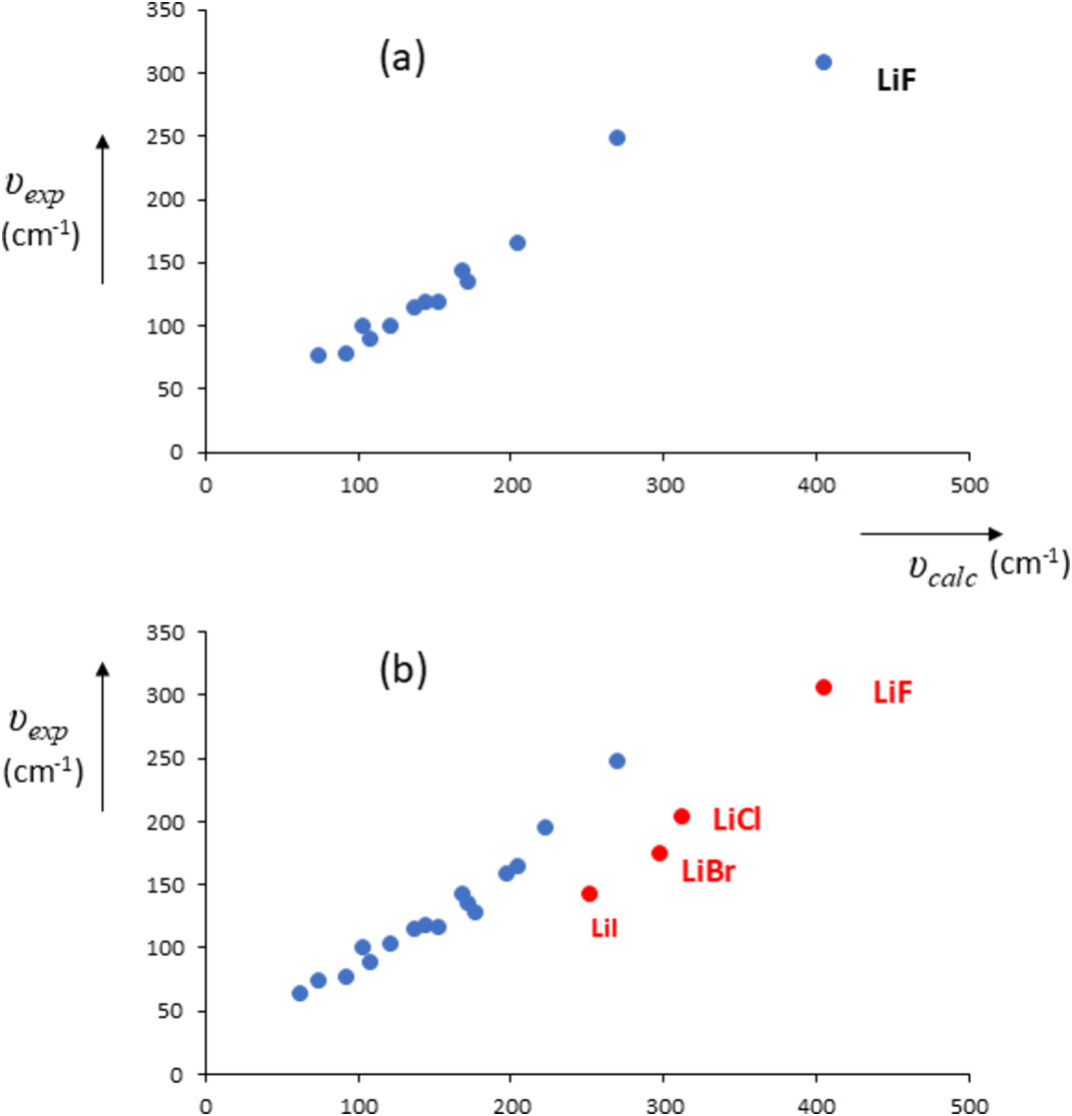
Experimental values for lattice vibrations *υ*_exp_ versus calculated values *υ*_calc_ by Huggins [97]: (a) experimental data from 1937; (b) present experimental data, lithium halides in red.

Finally, we discuss an early report (1937) by Huggins [97] which, in hindsight, appears highly relevant. This author published calculations on the “characteristic maximum infrared frequency,” which we equate to the transverse optical frequency. In Figure 14a, we compare his original calculations (from equations 9a and 9b in his work) with the experimental frequencies known at that time, but unfortunately, the experimental frequencies for LiCl, LiBr, and LiI were not known in 1937, and so the author could not distinguish these as a separate subset. In Figure 14b, we compare Huggins' calculations with the present experimental results for all alkali halides. We can now see that for all four lithium halides, the experimental frequencies are much smaller than the ones calculated by Huggins, which is precisely what would be expected from our analysis. We stress that these early calculations are remarkably accurate for the other halides and had all experimental values been known then, the lithium halides would undoubtedly have been found to have abnormally low lattice frequencies.

## 3 Summary

From comparative analyses of many bulk properties, it is concluded that the melting temperatures of the lithium halides are closely related to the quantity *μω* ^*2*^_*TO*_, where *μ* is the reduced mass of an ion pair and *ω*_*TO*_ is the lattice transverse optical (angular) frequency; *μω* ^*2*^_*TO*_ can be viewed as a combined force constant of the crystal. For the lithium halides, the transverse optical frequencies are considerably lower than expected from comparative considerations and we propose that the relatively low melting points for the lithium halides reflect these lower frequencies. The melting temperatures are also closely related to the dielectric constants *ɛ*, because *ɛ* and *ω*_*TO*_ are inter‐related; that is, low melting points correspond to high dielectric constants. This also follows from the diffusional force theory proposed by Neumann and Bosi. The Lindemann parameters for the lithium halides turn out to be exceedingly large (and constant for all lithium halides) and so the molecules “shake themselves loose” at relatively low temperatures. Our results do not invalidate earlier proposals for the low melting points of the lithium halides, but provide an alternative rationalization. It is further concluded that the dielectric constant of lithium iodide likely needs revision upwards from 11.03 to about 15.5.

## Author Contributions

**Lona Zeneyedpour:** methodology, analysis, and writing. **Peter C. Burgers:** data collection, methodology, analysis, and writing.

## Funding

The authors report no specific funding received for this study.

## Conflicts of Interest

The authors declare no conflicts of interest.

## Supporting information




**Data S1:** Calculation of Szigeti and Born charges (word‐document).

**Figure S1:** Melting points of the alkali halides as a function of the Szigeti and Born charges (word‐document).
**Figure S2:**. Static dielectric constant (*ɛ*) versus optical dielectric constant *ɛ∞* of the lithium halides. Blue: experimental values for LiF, LiCl and LiBr. Red: literature values for LiI. Green: estimated value *ɛ* (15.5 ± 0.8), this work (word‐document).

**Table S1:** Properties of alkali halides (excel document).

## Data Availability

The data supporting this article have been included as part of the Supporting Information.
