# Supplementary material for: Analysis of Melting Phenomena of the Alkali Halides: What Causes the Low Melting Points of the Lithium Halides?
Source: J Mass Spectrom. 2026 May 13;61:e70065. doi: 10.1002/jms.70065 (PMC13171234; doi:10.1002/jms.70065)
Supplement: Supplementary file 1 — Data S1: Calculation of Szigeti and Born charges (word‐document). [file JMS-61-e70065-s001.docx]

**Calculation of Szigeti and Born charges**

Regular frequencies (*υ*) are given in cm^-1^ throughout this paper. Then:

*µω*^2^ (Nm^-1^) = *µ*(2π*cυ*)^2^*N*^-1^**^.^**10^-3^ = 5.892**^.^**10^-5^*µυ*^2^ (*υ* in cm^-1^, *c* in cm**^.^**s^-1^, *µ* in u and *N* is Avogadro’s number)

**Szigeti charge s** (dimensionless number)

From angular frequency *ω*:

(sZ*e*)^2^ = 9*ɛ_0_Vµω*^2^*_TO_*(*ɛ*-*n*^2^)/(*n*^2^+2)^2^....(**19**) *µω*^2^*_TO_* in Nm^-1^, *V* in m^3^

From regular frequency *ν*:

(sZ*e*)^2^ = 36π^2^*ɛ_0_Vµc*^2^*υ*^2^*_TO_ N*^-1^(*ɛ*-*n*^2^)/(*n*^2^+2)^2^.10^-3^...(**19-1**) *υ_TO_* in cm^-1^, *µ* in u, *V* in m^3^, c in cms^-1^, 10^-3^ from g to kg

*c*^2^ = 10^11^(4π*ɛ_0_*)^-1^:

(sZ*e*)^2^ = 9π*Vµυ*^2^*_TO_ N*^-1^(*ɛ*-*n*^2^)/(*n*^2^+2)^2^.10^8^...(**19-2**) *υ_TO_* in cm^-1^, *µ* in u, *V* in m^3^

Hence Szigeti charge s:

**s = 4.277*υ_TO_*{*Vµ*(*ɛ*-*n*^2^)/(*n*^2^+2)^2^}^½^.10^-4^**....(**19-3**); *υ_TO_* in cm^-1^, *µ* in u, *V* in Å^3^, Z = 1, see Table S1 and Figure S1

**Born charge** **e^*^**(dimensionless number)

From angular frequency *ω*:

(e^*^Z*e*)^2^ = *ɛ_0_Vµω*^2^*_TO_*(*ɛ*-*n*^2^)....*µω*^2^*_TO_* in Nm^-1^

From regular frequency *ν*:

(e^*^Z*e*)^2^ = 4π^2^*ɛ_0_Vµc*^2^*υ*^2^*_TO_N*^-1^(*ɛ*-*n*^2^).10^-3^....*υ_TO_* in cm^-1^, *µ* in u, *V* in m^3^, c in cms^-1^, 10^-3^ from g to k

*c*^2^ = 10^11^(4π*ɛ_0_*)^-1^:

(e^*^Z*e*)^2^ = π*Vµυ*^2^*_TO_N*^-1^(*ɛ*-*n*^2^).10^8^....*υ_TO_* in cm^-1^, *µ* in u, *V* in m^3^

Hence Born charge e^*^:

**e^*^ = 1.426*υ_TO_*{*Vµ*(*ɛ*-*n*^2^)}^½^.10^-4^**; ....*υ_TO_* in cm^-1^, *µ* in u, *V* in Å^3^, Z = 1, see Table S1 and Figure S1
