# Supplementary material for: Analysis of Melting Phenomena of the Alkali Halides: What Causes the Low Melting Points of the Lithium Halides?
Source: J Mass Spectrom. 2026 May 13;61:e70065. doi: 10.1002/jms.70065 (PMC13171234; doi:10.1002/jms.70065)
Supplement: Supplementary file 2 — Figure S1: Melting points of the alkali halides as a function of the Szigeti and Born charges (word‐document). Figure S2:. Static dielectric constant (ɛ) versus optical dielectric constant ɛ∞ of the lithium halides. Blue: experimental values for LiF, LiCl and LiBr. Red: literature values for LiI. Green: estimated value ɛ (15.5 ± 0.8), this work (word‐document). [file JMS-61-e70065-s003.docx]

**Supplementary Figures**


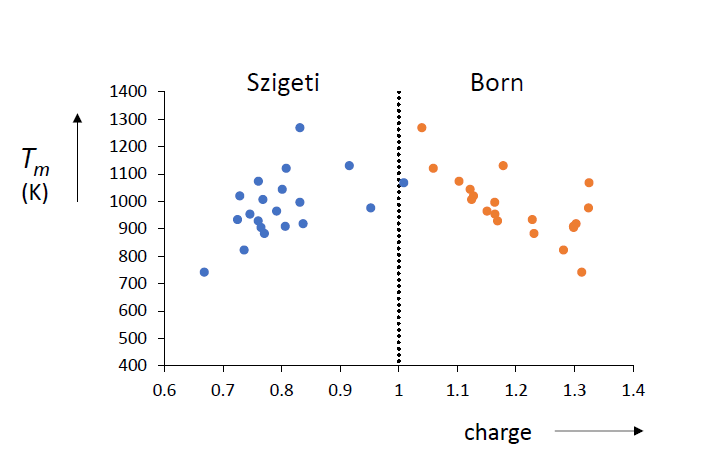


Figure S1. Melting points of the alkali halides as a function of the Szigeti and Born charges


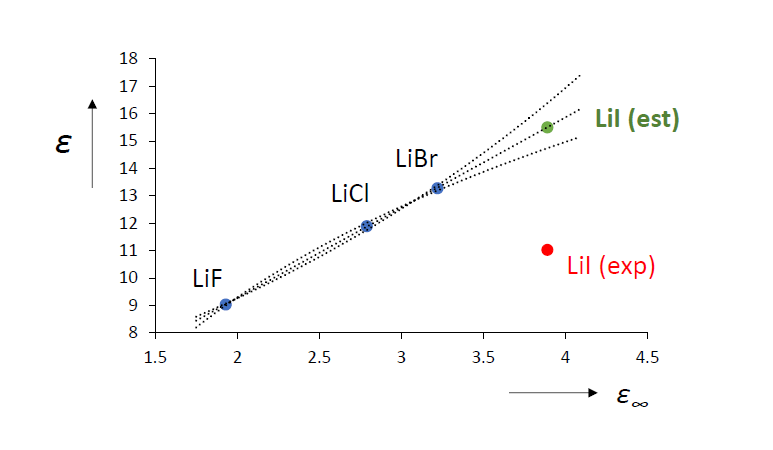


Figure S2. Static dielectric constant (*ɛ*) versus optical dielectric constant *ɛ_∞_* of the lithium halides. Blue: experimental values for LiF, LiCl and LiBr. Red: literature values for LiI. Green: estimated value *ɛ* (15.5 ± 0.8), this work
